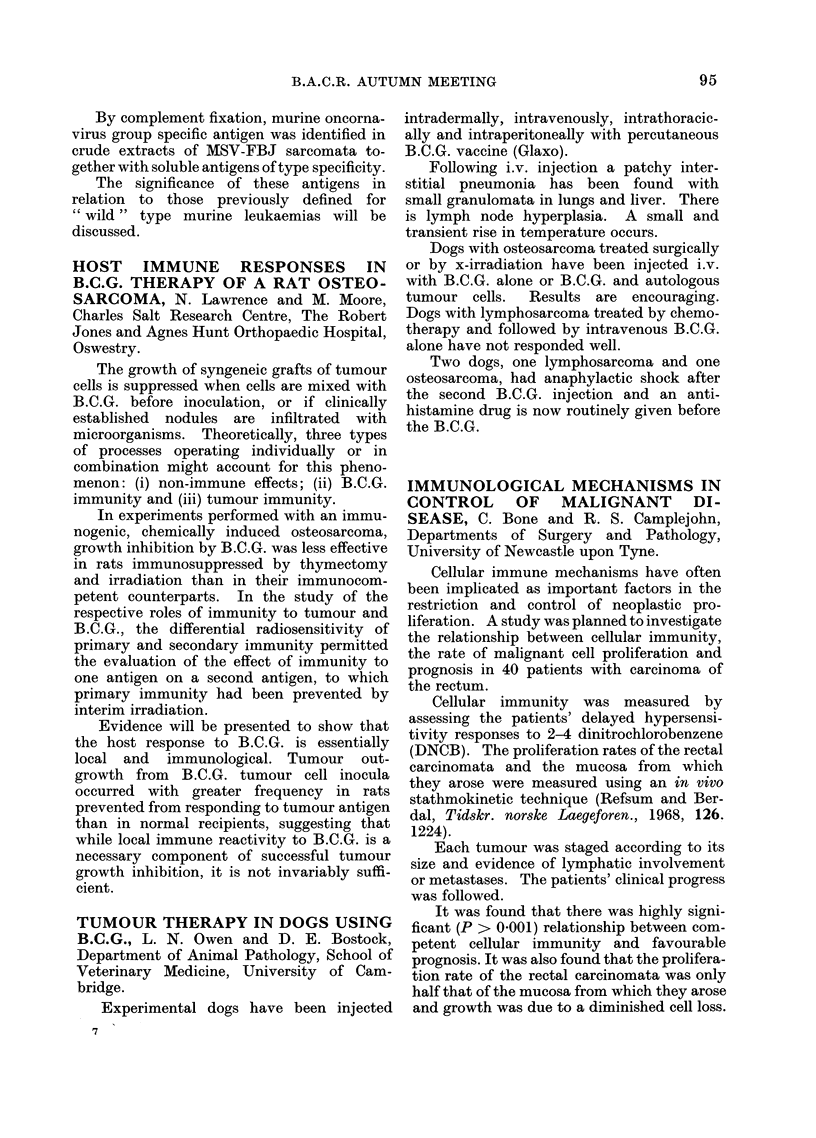# Proceedings: Tumour therapy in dogs using B.C.G.

**DOI:** 10.1038/bjc.1974.24

**Published:** 1974-01

**Authors:** L. N. Owen, D. E. Bostock


**TUMOUR THERAPY IN DOGS USING B.C.G.,** L. N. Owen and D. E. Bostock, Department of Animal Pathology, School of Veterinary Medicine, University of Cambridge.

Experimental dogs have been injected intradermally, intravenously, intrathoracically and intraperitoneally with percutaneous B.C.G. vaccine (Glaxo).

Following i.v. injection a patchy interstitial pneumonia has been found with small granulomata in lungs and liver. There is lymph node hyperplasia. A small and transient rise in temperature occurs.

Dogs with osteosarcoma treated surgically or by x-irradiation have been injected i.v. with B.C.G. alone or B.C.G. and autologous tumour cells. Results are encouraging. Dogs with lymphosarcoma treated by chemotherapy and followed by intravenous B.C.G. alone have not responded well.

Two dogs, one lymphosarcoma and one osteosarcoma, had anaphylactic shock after the second B.C.G. injection and an antihistamine drug is now routinely given before the B.C.G.